# Are a Child’s Autistic Traits, Behavioural Difficulties, Prosocial Behaviour and Temperament Predictors of Parental Self-Efficacy and Satisfaction? A Study on Parents of Autistic and Neurotypical Children Aged 7–11 Years

**DOI:** 10.1007/s10803-024-06517-w

**Published:** 2024-08-28

**Authors:** Iwona Omelańczuk, Ewa Pisula

**Affiliations:** 1 Institute of Psychology, The Maria Grzegorzewska University, Warsaw, Poland; 2 Faculty of Psychology, University of Warsaw, Warsaw, Poland

**Keywords:** Parental self-efficacy, Parental satisfaction, Autistic traits, Behavioural difficulties, Prosocial behaviour, Temperamental characteristics

## Abstract

The aim of the study was to evaluate the significance of the severity of autistic traits, behavioural difficulties, prosocial behaviour and temperamental characteristics in children for parental self-efficacy and parental satisfaction in two groups of parents: parents of autistic children, and parents of neurotypical children. Data come from 145 parents of autistic children and 239 parents of neurotypical children. Using hierarchical multiple regression analyses, the analysis explored the role of child characteristics in prediction of parental self-efficacy and parental satisfaction. The regression model tested explained 21% variation in parental self-efficacy and 27% variation in parental satisfaction in parents of autistic children and 3% of variation of results with respect to parental self-efficacy and 17% variation in parental satisfaction in parents of neurotypical children. In both groups, parental self-efficacy and parental satisfaction were negatively correlated with such child characteristics as severity of behavioural difficulties, severity of autistic traits and emotionality as also positively related to the child’s prosocial behaviour. These findings may suggest that particularly useful mental health prevention programs for parents should combine two elements: developing parents’ abilities of effectively coping with children’s behavioural difficulties and working on attribution processes and negative convictions about parenthood.

The concept of parental self-efficacy (PSE) refers to the way parents perceive their own ability to perform various childcare-related tasks and the degree to which they feel competent and confident in performing those tasks (Jones & Prinz, 2005). PSE consists of several components, including a parent’s belief in their own ability to achieve desired results in parenting the child, subjective assessment of their own knowledge about appropriate parenting behaviour and the level of confidence in their ability to perform their parenting role successfully (Coleman & Karraker, 1998).

Parental self-efficacy is considered to be one of the key factors determining the child’s development (Albanese et al., 2019). Parents with high PSE are more likely to solve parenting problems by encouraging collaboration in their child (Ardelt et al., 2001), are more responsive to the child’s needs (Abuhammad, 2021) and more effective in supporting the child’s all-round development (Kuhn & Carter, 2006; Meunier et al., 2010). High PSE has positive implications also for the psychological well-being of parents (Albanese et al., 2019; Feng et al., 2022). Parents who feel confident in caring for and raising their children declare lower severity of depressive symptoms (Kohlhoff & Barnett, 2013) and lower levels of parenting stress (Higgins et al., 2022).

Parental self-efficacy is closely associated with parental satisfaction, which involves the quality of parenting-related emotions, especially the sense of pleasure derived from performing the role of parent (Coleman & Karraker, 1998; Jones & Prinz, 2005). An important aspect of that process might be the fact that high-PSE parents tend to view parenting difficulties as challenges to overcome rather than threats to be avoided. Expecting to succeed in parental tasks helps deal with them and draw satisfaction and pleasure from being a parent (Erdwins et al., 2001; Finzi-Dottan et al., 2011).

As reported by Fang et al. (2021), out of all studies on the factors associated with PSE conducted between 1980 and 2020, only 12% evaluated child characteristics. Children’s developmental problems are known to potentially play an important role in the context of PSE, with parents of autistic children being particularly at risk for low PSE and low parental satisfaction (Jankowska et al., 2022; Lau & Peterson, 2011). These parents face challenges resulting from the complex and unique needs of their children (Schertz et al., 2020), often taking on the double role of parent and therapist (Safe et al., 2012). However, as demonstrated in the review of research by Silva and Roama-Alves (2023), there have still been very few studies on PSE in parents of autistic children. What is more, analyses mainly focus on the effectiveness of parental/child intervention programs or parental cognition and outcomes. Thus, it remains unclear which the characteristics of an autistic child may play a significant role in the prediction of PSE and parental satisfaction.

Most analyses suggest that children’s demographic characteristics (age or gender), are unrelated to PSE or parental satisfaction both in the parents of autistic (Taylor et al., 2021; Weiss et al., 2015) and neurotypical (Fang et al., 2021; Glatz et al., 2023) children. The important question is whether the severity of autistic symptoms that include difficulties in social communication and social interactions, as well as restricted patterns of behaviour, interests and activities (APA, 2013), is significant for PSE and parental satisfaction. So far, relationships between these variables were investigated in a handful of studies focused on families with autistic children and their results are mixed. Some analyses showed negative relationship between autism symptoms severity in child and PSE (Rezendes & Scarpa, 2011; Taylor et al., 2021; Weiss et al., 2015) or parental satisfaction (Falk et al., 2014), while others found no statistically significant relationship between these variables, i.e. PSE (Garcia-Lopez et al., 2016; Kurzrok et al., 2021; Lindsey & Barry, 2018; Russell & Ingersoll, 2021) and parental satisfaction (Tobing & Glenwick, 2007).

Maybe, among the key characteristics associated with PSE and parental satisfaction are the child’s behavioural and emotional difficulties. As reported by Glatz et al. (2023) in the review of studies on parental self‐efficacy among parents of neurotypical children, higher severity of externalizing (e.g. disobedience toward adult, disruptiveness, antisocial behaviours, hyperactivity–distractibility or being angry, aggressive and egoistic) and internalizing (e.g. depressed mood, anxiety and fear or loneliness) behavioural difficulties in children are associated with lower PSE. A negative relationship between behavioural and emotional difficulties and parental satisfaction was also found (Matalon & Turliuc, 2022; Salari et al., 2014).

However, the results of studies examining the relationship between behavioural difficulties in autistic children and PSE or parental satisfaction are also not consistent. Some of the analyses found a positive correlation between these variables (Chen et al., 2021; Kabashima et al., 2020; Lindsey & Barry, 2018) and a predictive role of child’s behavioural difficulties for PSE (Rudelli et al., 2021). This seems clear, given that the higher the severity of the child’s behavioural difficulties, the more difficult it is to perform parenting tasks and the lower are the chances of achieving desired results in raising the child. In addition, the child’s behavioural difficulties mean that parents are less favourably perceived and evaluated in their roles by others (Gray, 2002). Difficulties encountered by the parent, often exacerbated by criticism from others, impinge on parental beliefs about their self-efficacy and affect the emotions experienced in performing the role of parent (Hodgetts et al., 2013; Myers et al., 2009). However, these relationships may be more complex. Giallo et al. (2011) found that behavioural difficulties in autistic children were connected with parental satisfaction but not with PSE. Lack of association between challenging behaviour in autistic children and PSE was also shown in other studies (Benson & Kersh, 2011; Rezendes & Scarpa, 2011; Taylor et al., 2021). Moreover, some studies indicated no statistically significant relationships between parental satisfaction and behavioural difficulties in autistic children (Rudelli et al., 2021). It is difficult to unequivocally explain the discrepancies between the results of these studies. Some additional data may come from analysing the child’s temperamental traits, which is a factor associated with behavioural difficulties in children (Coe et al., 2020; Harvey et al., 2022).

Jones and Prinz (2005) suggested that not only the behavioural difficulties but also the positive behaviours of a child are related to PSE or parental satisfaction. One of these strengths is a child’s prosocial behaviour, and parents whose neurotypical children engage in actions such as sharing, comforting, or helping others, feel more effective in the role of parent (Luengo Kanacri et al., 2021; Trecca et al., 2022; Woolgar et al., 2023). Also, a child’s school-related social competence (cooperating skills, empathy, impulsivity and disruptiveness) is positively correlated with parental self-efficacy (Junttila & Vauras, 2014; Junttila et al., 2007). Unfortunately, according to a systematic review by Ryan-Enright et al. (2022), prosocial behaviour is rarely analysed in autistic children, so little is known about the relationships between this type of behaviour in this group of children and PSE or parental satisfaction. However, a positive relationship between prosocial behaviour in autistic children and PSE was found (Benedetto et al., 2021; Benson, 2014).

Temperamental characteristics in neurotypical children are related to parental behaviours, parental beliefs about their own effectiveness and enjoyment in the carrying out of parenting tasks (Jones & Prinz, 2005; Kiff et al., 2011). Parents whose children respond to environmental events with strong negative emotions declare lower pleasure of being a parent (Coleman & Karraker, 2000), and lower PSE (Lipscomb et al., 2011; Solmeyer & Feinberg, 2011). Among the child’s temperamental characteristics particularly important for PSE is emotionality: the higher it is, the lower the level of PSE (Coleman & Karraker, 2000). Lower PSE is also reported in parents whose children are characterized by less sociability (Coleman & Karraker, 2000) and soothability (Leerkes & Crockenberg, 2002; Verhage et al., 2013). Also, difficult temperament, as described by Thomas and Chess (1981), characterized by irregularity of biological functions, tendency to withdraw, slow adaptation to changes, high intensity of affect expression and frequent negative and rare positive mood, is connected with lower PSE (Porter & Hsu, 2003; Sevigny & Loutzenhiser, 2010), and is one of the temperamental predictors of PSE (Giallo et al., 2013; Machida et al., 2002). Unfortunately, out of the entire body of research conducted from 1980 to 2020 on factors associated with PSE, only 3.3% looked at the child’s temperament (Fang et al., 2021). What is more, the vast majority of those studies analysed the relationship between PSE and temperament in young children, i.e. infants (Lipscomb et al., 2011; Solmeyer & Feinberg, 2011; Verhage et al., 2013) or toddlers (Giallo et al., 2013; Machida et al., 2002; Sevigny & Loutzenhiser, 2010). According to a recent systematic review by Mallise et al. (2020), although the temperament in autistic children is frequently analysed, there is still little information about its relationship to PSE or parental satisfaction.

The aim of this study was to examine relationships between parental self-efficacy or parental satisfaction and such characteristics of the child as severity of autistic traits, behavioural difficulties, prosocial behaviour, and temperamental characteristics in two groups of parents, i.e., parents of autistic children and parents of neurotypical children aged 7–11. Moreover, we evaluated the role of these characteristics in the child in the prediction of parental self-efficacy and parental satisfaction in parents of autistic children and parents of neurotypical children. We hypothesized that both PSE and parental satisfaction would be negatively related to the severity of autistic traits, behavioural difficulties and emotionality in children and positively associated with the child’s prosocial behaviour.

According to the autistic traits hypothesis (Baron-Cohen et al., 2001), the features of social, communicative and cognitive function typical for autism, as well as the tendency for restricted, repetitive behaviours and interests may be treated as quantitative characteristics, distributed continuously throughout the population. In this view, individuals meeting the criteria for a clinical diagnosis of autism spectrum disorder demonstrate extreme severity of autistic traits, but the traits themselves are present to some degree in neurotypical individuals as well (Constantino & Todd, 2003; Sucksmith et al., 2011). So far, we have limited knowledge about the significance of the severity of these traits in neurotypical children for the functioning of their parents. To the best of our knowledge, this topic not been empirically examined, which means that obtaining these data would provide new information both about the factors affecting PSE and parental satisfaction both in the parents of autistic and neurotypical children.

As mentioned above, the vast majority of information about the relationship between neurotypical children’s characteristics, especially temperament, and PSE or parental satisfaction, comes from research on parents of young children (≤ 6 years of age) (Machida et al., 2002; Solmeyer & Feinberg, 2011). Also, the relationship between the child’s prosocial behaviour and parenting is analysed mainly in the context of parenting practice, warmth or involvement (van der Storm et al., 2022; Wong et al., 2021). So, there is still a gap regarding the relationship between prosocial behaviour in children and parental self-efficacy or satisfaction. What’s more, research on parents of autistic children often involves groups that are highly heterogeneous in terms of the age of children (Falk et al., 2014; Kurzrok et al., 2021; Rudelli et al., 2021) and comorbidity of the autism spectrum disorder with intellectual disabilities or other conditions (Lindsey & Barry, 2018; Rezendes & Scarpa, 2011; Weiss et al., 2015). The wide range of autistic children’s age and level of intellectual functioning may affect parental self-efficacy and satisfaction, complicating the interpretation of data on the determinants of these parental characteristics. The current study will involve parents of children without intellectual disabilities aged from 7 to 11 years. We know very little about the situation of this group in terms of PSE and parental satisfaction. At 6–7 years of age children in Poland begin formal school education. This is an important milestone in the life of the child and parent, one that comes with a number of new challenges and requirements. Children have to adapt to new circumstances and meet new cognitive, emotional and social challenges, which might give parents the sense that their effectiveness as caregivers is being judged (Connolly & Gersch, 2016).

## Methods

### Ethical Considerations

This study was conducted by the guidelines of the Declaration of Helsinki. The approval of the Ethical Commission of the Faculty of Psychology, University of Warsaw, was obtained for this study. All participants gave informed consent before they participated in the study.

### Procedure

This was a questionnaire-based, pen-and-paper study. Participants were recruited throughout Poland via educational institutions their children attended (mainstream and inclusive schools or school for autistic children). Invitations to take part in the study were sent to 97 schools, 65 of which responded positively by forwarding the information about the study to interested parents. In the first step, parents received general information about the purpose and nature of the study through the schools. Those potentially interested in participating in the study were asked to contact the researchers via e-mail for more detailed information. Those who finally decided to participate were given a set of questionnaires and an envelope with a return address. Parents were asked to think of one child and complete all child-related questionnaires (including a demographic survey) assessing this particular child. They were also asked to fill out a questionnaire about parenting in relation to this specific child. Out of the 1958 sets of instruments sent out in total, 648 (33%) were sent back.

### Participants

The analysis included data from 145 parents of autistic children (Autistic Group) and 239 parents of neurotypical children (Neurotypical Group)—it was the mother or the father of the child, never the mother and the father of the same child. Educational attainment level of parents was from primary to higher, but family income level was not recorded. The children’s age ranged from 7 to 11 years, all children attended either mainstream or inclusive schools or school for autistic children. All children in the Autistic Group had a formal psychiatric diagnosis based on ICD-10 criteria (WHO, 1992), independently from this study. The parents of children with childhood autism (n = 64), Asperger syndrome (n = 61) and atypical autism (n = 20) were involved.

The study did not include data from 76 parents of autistic children due to missing data in the questionnaires (n = 34), the age of the child over 11 years (n = 3), or additional diagnoses in autistic children: intellectual disabilities, attention deficit hyperactivity disorder, obsessive–compulsive disorder, epilepsy, asthma, etc. (n = 39). The parents of neurotypical children were included to the Neurotypical Group based on their declaration that the child had no diagnosis of developmental problems or chronic illness and was not involved in an ongoing or planned diagnostic process. The analysis did not include data from 137 parents of neurotypical children due to missing data in the questionnaires (n = 116), the age of the child over 11 years (n = 6), and the child’s diagnosis of attention deficit hyperactivity disorder (n = 11) or dyslexia (n = 4). The parents whose neurotypical children scored above the cut-off point for the Polish version of AQ-Child (score above 67) were also excluded from the study (*n* = 51).

The demographic characteristics of the Autistic Group and the Neurotypical Group are provided in Table 1.

**Table 1:** Demographic characteristics of the Autistic Group and the Neurotypical Group

		Autistic Group N = 145	Neurotypical Group n = 239
Parent’s gender	Female	n = 134	n = 216
	Male	n = 11	n = 23
Parent’s age		M = 38.86 SD = 5.37	M = 37.73 SD = 4.84
Parent’s education	Primary	0%	1.4%
	Vocational	4.1%	2.9%
	Secondary	37.3%	23.8%
	Higher	58.6%	71.9%
Child’s gender	Girl	n = 17	n = 153
	Boy	n = 128	n = 86
Child’s age		M = 8.66 SD = 1.30	M = 8.46 SD = 1.33

In terms of demographic characteristics there was a statistically significant difference between the groups in parent age (*t*(378) = − 2.104; *p* = 0.036); the parents in the Autistic Group were older than the parents in the Neurotypical Group. However, the age difference between the two groups was not large (Cohen’s *d* = 0.22). Group differences were also apparent in the children’s gender distribution (χ^2^(1) = 100.026; *p* < 0.001). In the Autistic Group, there were more boys than in the Neurotypical Group. This is consistent with epidemiological reports that autism spectrum disorder is diagnosed several times more frequently in boys than in girls (Maenner et al., 2023).

### Measures

*Parental self-efficacy and parental satisfaction* were measured with the Parenting Sense of Competence Scale, PSoC*;* original version: Johnston & Mash, 1989; Polish language adaptation Jankowska et al., 2022. This is a 16-item self-report measure. The items make up two subscales: the parental self-efficacy scale and the parental satisfaction scale. Higher scores indicate higher parental self-efficacy or higher parental satisfaction. In this study, the value of Cronbach’s alpha reliability coefficient was α = 0.746 for parental self-efficacy and α = 0.76 for parental satisfaction in the Autistic Group and α = 0.739 for parental self-efficacy and α = 0.789 for parental satisfaction in the Neurotypical Group.

*Autistic traits* in children were measured using The Autism Spectrum Quotient: Children’s Version (AQ–Child, Auyeung et al., 2008; Polish language adaptation, article in press). The questionnaire consists of 50 items (answers are scored from 0 to 3) that make up five subscales: social skills, attention to detail, attention switching, communication and imagination. The present study used the total score, indicating the severity of autistic traits in the child. The total score ranges from 0 (no autistic traits) to 150 (extreme severity of autistic traits). Internal reliability of AQ–Child was found to be α = 0.917 for the Autistic Group and α = 0.747 for the Neurotypical Group.

*Behavioural difficulties* and *prosocial behaviour* were assessed using the Strengths and Difficulties Questionnaire (SDQ; Goodman, 1997). The Polish version of this parent-rated measure prepared by Mazur et al. (2007) was used. SDQ comprises 25 items categorised into 5 subscales: emotional problems, conduct problems, hyperactivity, peer problems and prosocial behaviour. Summed scores from all subscales, except prosocial behaviour, give the total difficulties score. Higher scores indicate higher severity of behavioural difficulties or greater tendency to prosocial behaviour. In the study, Cronbach’s alpha in the Autistic Group for total behavioural difficulties score was α = 0.734 and α = 0.772 for prosocial behaviour. Cronbach’s alpha reliability coefficients in the Neurotypical Group were as follows: α = 0.769 for total behavioural difficulties score and α = 0.71 for prosocial behaviour.

*Child temperament* was measured using the Emotionality, Activity, and Sociability Temperament Survey: The Parent-rated Version (EAS-C; Buss & Plomin, 1984; in Polish adaptation by Oniszczenko, 1997). The questionnaire consists of 20 items (5 items for each dimension of temperament: emotionality, activity, sociability and shyness), with higher scores indicating greater severity of a given dimension of temperament. Cronbach’s alpha reliability coefficients for each temperamental dimension in the Autistic Group were as follows: α = 0.559 for emotionality, α = 0.79 for activity, α = 0.77 for sociability and α = 0.767 for shyness. In the Neurotypical Group, Cronbach’s alphas for individual temperamental dimensions were: α = 0.622 for emotionality, α = 0.76 for activity, α = 0.518 for sociability and α = 0.727 for shyness.

### Data Analysis

Data were screened for normality, using skewness and kurtosis values. All ranges of skewness and kurtosis were acceptable for normal distribution.

Pearson’s r correlation analysis was used to examine correlations between parental self-efficacy or parental satisfaction and child characteristics. Parental self-efficacy and parental satisfaction predictors were tested using hierarchical multiple regression analyses. In the first step, the child’s demographic characteristics (gender and age) were added into the regression models. Then, the variables were entered into the regression models considering the strength of correlation between parental self-efficacy or satisfaction and the child’s other characteristics in each group separately. All analyses were performed using SPSS 29.0.

## Results

Table 2 presents descriptive statistics for the eight study measures in the Autistic Group and the Neurotypical Group.

**Table 2:** Descriptive statistics for the all measures in the Autistic Group and the Neurotypical Group

Variables	Autistic Group (*N* = 145)		Neurotypical Group (*N* = 239)	
	*M* (*SD*)	Range	*M* (*SD*)	Range
Parent characteristic				
Parental self-efficacy	28.27 (5.12)	17–41	30.11 (4.68)	17–41
Parental satisfaction	36.43 (7.13)	17–52	40.08 (6.99)	15–53
Child characteristic				
Autistic traits	88.83 (19.77)	31–136	52.13 (9.70)	19–66
Behavioural difficulties	17.94 (5.45)	7–31	8.27 (4.73)	0–25
Prosocial behaviour	5.67 (2.40)	0–10	8.47 (1.65)	1–10
Emotionality	17.26 (3.51)	9–25	14.72 (3.34)	8–24
Activity	17.56 (4.71)	8–25	20.46 (3.40)	8–25
Sociability	14.23 (4.37)	5–24	19.57 (2.79)	10–25
Shyness	14.35 (4.59)	5–24	10.18 (3.37)	5–22

Results of Pearson’s *r* correlation analyses are presented in Table 3.

**Table 3:** Pearson’s r correlation between parental self-efficacy, parental satisfaction and characteristics of child in the Autistic Group and the Neurotypical Group

Variables	Parental self-efficacy		Parental satisfaction	
	Autistic Group (*N* = 145)	Neurotypical Group (*N* = 239)	Autistic Group (*N* = 145)	Neurotypical Group (*N* = 239)
Autistic traits	− 0.28***	− 0.09	− 0.20*	− 0.23***
Behavioural difficulties	− 0.43***	− 0.15*	− 0.45***	− 0.37***
Prosocial behaviour	0.37***	0.14*	0.43***	0.28***
Emotionality	− 0.23**	− 0.05	− 0.27***	− 0.26***
Activity	0.13	0.08	− 0.01	− 0.01
Sociability	0.23**	− 0.02	0.13	− 0.01
Shyness	− 0.22**	0.04	− 0.15	− 0.08

*p < 0.05; **p < 0.01; ***p < 0.001

Results of hierarchical multiple regression analyses for PSE as the explained variable are presented in Table 4 (Autistic Group) and Table 5 (Neurotypical Group).

Hierarchical multiple regression analyses for parental self-efficacy were conducted in five steps in the Autistic Group. In the first step, the demographic characteristics of the child (gender and age) were fed into the model. The model was not statistically significant (Δ*F*(2, 142) = 0.316; *p* = 0.730). In step two, the severity of behavioural difficulties in the child was added to the regression model, resulting in a statistically significant increase in the percentage of explained variance in the scores on parental self-efficacy at 18% (Δ*F*(1, 141) = 30.559; *p* < 0.001). The severity of behavioural difficulties was the only predictor of parental self-efficacy (β = − 0.43; *p* < 0.001). The model explained 18% of score variance in parental self-efficacy in the Autistic Group. In the third step, the model was fed with prosocial behaviour in the child, and this model was statistically significant (Δ*F*(1, 140) = 4.592 *p* = 0.034). Significant predictors were the severity of behavioural difficulties (β = − 0.32; p < 0.001) and prosocial behaviour (β = 0.19; *p* = 0.034) in the child. The model explained 21% of score variance in parental self-efficacy in the Autistic Group. In step four, the model was fed with the severity of autistic traits, and in step five it was fed with three predictors in the form of the child’s temperamental characteristics. Following that addition, the change in the percentage of the explained score variance in parental self-efficacy in the Autistic Group proved to be insignificant both in step 4 (Δ*F*(1, 139) = 0.212; *p* = 0.646) and in step 5 (Δ*F*(3, 136) = 0.749; *p* = 0.525) (Table 4).

**Table 4:** Regression models for measures of child characteristic, as predictors of parental self-efficacy for the Autistic Group

Autistic Group (*N* = 145)					
	*B (SE)*	β	*R*^*2*^	Δ*R*^*2*^	VIF
Step 1					
			0.00	0.00	
Child’s gender	0.41 (1.33)	0.03			1.01
Child’s age	− 0.23 (0.33)	− 0.06			1.01
Step 2					
			0.18***	0.18***	
Child’s gender	0.35 (1.21)	0.02			1.01
Child’s age	0.06 (0.31)	0.01			1.04
Behavioural difficulties	− 0.40 (0.07)	− 0.43***			1.03
Step 3					
			0.21*	0.03*	
Child’s gender	0.25 (1.20)	0.02			1.01
Child’s age	0.01 (0.30)	− 0.00			1.04
Behavioural difficulties	− 0.30 (0.09)	− 0.32***			1.49
Prosocial behaviour	0.41 (0.19)	0.19*			1.45
Step 4					
			0.21	0.00	
Child’s gender	0.28 (1.20)	0.02			1.01
Child’s age	0.02 (0.30)	− 0.01			1.05
Behavioural difficulties	− 0.29 (0.09)	− 0.31**			1.60
Prosocial behaviour	0.38 (0.20)	0.18			1.62
Autistic traits	− 0.01 (0.02)	− 0.05			1.45
Step 5					
			0.22	0.01	
Child’s gender	− 0.11 (1.21)	− 0.01			1.02
Child’s age	0.10 (0.31)	0.03			1.10
Behavioural difficulties	− 0.29 (0.10)	− 0.31**			2.15
Prosocial behaviour	0.37 (0.21)	0.17			1.65
Autistic traits	0.01 (0.03)	0.05			2.17
Emotionality	− 0.03 (0.13)	− 0.02			1.47
Sociability	0.06 (0.13)	0.05			2.00
Shyness	− 0.12 (0.11)	− 0.11			1.73

*p < 0.05; **p < 0.01; ***p < 0.001

Hierarchical multiple regression analyses for parental self-efficacy were conducted in 3 steps in the Neurotypical Group. In the first step the demographic characteristics of the child (gender and age) were fed into the model. The model was not statistically significant (Δ*F*(2, 236) = 0.207; *p* = 0.814). In step 2, the severity of behavioural difficulties in the child was added to the regression model, resulting in a statistically significant increase in the percentage of explained variance in the scores on parental self-efficacy at 3% (Δ*F*(1, 235) = 5.820; *p* = 0.017). The severity of behavioural difficulties was the only predictor of parental self-efficacy (β = − 0.16; *p* = 0.017). The model explained 3% of score variance in parental self-efficacy in the Neurotypical Group. In third step, the model was fed with prosocial behaviour in the child. Following that addition, the change in the percentage of explained score variance in parental self-efficacy in the Neurotypical Group proved insignificant (Δ*F*(1, 234) = 1.779; *p* = 0.184) (Table 5).

**Table 5:** Regression models for measures of child characteristic, as predictors of parental self-efficacy for the Neurotypical Group

Neurotypical Group (*N* = 239)					
	*B (SE)*	β	*R*^*2*^	Δ*R*^*2*^	VIF
Step 1					
			0.00	0.00	
Child’s gender	− 0.12 (0.63)	− 0.01			1.00
Child’s age	− 0.14 (0.23)	− 0.04			1.00
Step 2					
			0.03*	0.03*	
Child’s gender	− 0.44 (0.64)	− 0.05			1.05
Child’s age	− 0.15 (0.23)	− 0.04			1.00
Behavioural difficulties	− 0.16 (0.07)	− 0.16*			1.05
Step 3					
			0.04	0.01	
Child’s gender	− 0.45 (0.64)	− 0.05			1.05
Child’s age	− 0.12 (0.23)	− 0.04			1.01
Behavioural difficulties	− 0.12 (0.07)	− 0.12			1.23
Prosocial behaviour	0.27 (0.20)	0.09			1.20

^*^p < 0.05; **p < 0.01; ***p < 0.001

Results of multiple regression analyses for parental satisfaction as the explained variable are presented in Table 6 (Autistic Group) and Table 7 (Neurotypical Group).

To identify the predictors of parental satisfaction, hierarchical multiple regression analyses were conducted in 5 steps for both groups. In the first step, demographic characteristics of the child (gender and age) were entered into the model. The model proved to be insignificant both in the Autistic Group (Δ*F*(2, 142) = 1.047; *p* = 0.354) and in the Neurotypical Group (Δ*F*(2, 236) = 1.160; *p* = 0.315). In step 2, the severity of the child’s behavioural difficulties was added to the regression model. The model explained 23% of the variance in parental satisfaction in the Autistic Group (Δ*F*(1, 141) = 38.537; *p* < 0.001) and 17% in the Neurotypical Group (Δ*F*(1, 235) = 44.002; *p* < 0.001). The statistically significant predictor in the Autistic Group (β = − 0.47; *p* < 0.001) and in the Neurotypical Group (β = − 0.40; *p* < 0.001) was the severity of behavioural difficulties in the child. In the following, third, step, prosocial behaviour in the child was added to the regression model. This model was significant only in the Autistic Group (Δ*F*(1, 140) = 7.684; *p* = 0.006). Significant predictors were the child’s severity of behavioural difficulties (β = − 0.33; p < 0.001) and prosocial behaviour (β = 0.24; *p* = 0.006). The model explained 27% of variance in parental satisfaction in the Autistic Group. In the Neurotypical Group, the model was not statistically significant (Δ*F*(1, 234) = 1.921; *p* = 0.167). In step four, the model was fed with the child’s emotionality. Following that addition, the change in the percentage of explained score variance in parental satisfaction proved to be insignificant both in the Autistic Group (Δ*F*(1, 139) = 0.498; *p* = 0.481) and in the Neurotypical Group (Δ*F*(1, 233) = 2.455; *p* = 0.119). In the last step, the severity of autistic traits in the child was added to the model. The model was not statistically significant in the Autistic Group (Δ*F*(1, 138) = 1.268; *p* = 0.261). This model was also insignificant in the Neurotypical Group (Δ*F*(1, 232) = 2.862; *p* = 0.092) (Table 6 and Table 7).

**Table 6:** Regression models for measures of child characteristic as predictors of parental satisfaction for the Autistic Group

Autistic Group (*N* = 145)					
	*B (SE)*	β	*R*^*2*^	Δ*R*^*2*^	VIF
Step 1					
			0.02	0.02	
Child’s gender	2.67 (1.85)	0.12			1.01
Child’s age	0.09 (0.46)	− 0.02			1.01
Step 2					
			0.23***	0.21***	
Child’s gender	2.58 (1.64)	0.12			1.01
Child’s age	0.53 (0.41)	0.10			1.04
Behavioural difficulties	− 0.61 (0.10)	− 0.47***			1.03
Step 3					
			0.27**	0.04**	
Child’s gender	2.40 (1.61)	0.11			1.01
Child’s age	0.45 (0.41)	0.08			1.04
Behavioural difficulties	− 0.43 (0.12)	− 0.33***			1.49
Prosocial behaviour	0.72 (0.26)	0.24**			1.45
Step 4					
			0.27	0.00	
Child’s gender	2.43 (1.61)	0.11			1.01
Child’s age	0.46 (0.41)	0.08			1.04
Behavioural difficulties	− 0.37 (0.13)	− 0.30**			2.00
Prosocial behaviour	0.73 (0.26)	0.25**			1.46
Emotionality	− 0.13 (0.18)	− 0.06			1.45
Step 5					
			0.28	0.01	
Child’s gender	2.35 (1.60)	0.11			1.01
Child’s age	0.43 (0.41)	0.08			1.05
Behavioural difficulties	− 0.43 (0.14)	− 0.33**			2.15
Prosocial behaviour	0.83 (0.27)	0.28**			1.62
Emotionality	− 0.11 (0.18)	− 0.06			1.45
Autistic traits	0.04 (0.03)	0.10			1.46

*p < 0.05; **p < 0.01; ***p < 0.001

**Table 7:** Regression models for measures of child characteristic as predictors of parental satisfaction for the Neurotypical Group

Neurotypical Group (*N* = 239)					
	*B (SE)*	β	*R*^*2*^	Δ*R*^*2*^	VIF
Step 1					
			0.01	0.01	
Child’s gender	− 1.16 (0.90)	− 0.08			1.01
Child’s age	0.28 (0.33)	0.06			1.01
Step 2					
			0.17***	0.16***	
Child’s gender	− 2.32 (0.85)	− 0.17			1.05
Child’s age	0.26 (0.30)	0.05			1.00
Behavioural difficulties	− 0.57 (0.09)	− 0.40***			1.05
Step 3					
			0.17	0.00	
Child’s gender	− 2.34 (0.85)	− 0.17			1.05
Child’s age	0.29 (0.30)	0.06			1.00
Behavioural difficulties	− 0.52 (0.09)	− 0.37***			1.23
Prosocial behaviour	0.37 (0.26)	0.09			1.19
Step 4					
			0.18	0.01	
Child’s gender	− 2.18 (0.85)	− 0.16			1.06
Child’s age	0.30 (0.30)	0.06			1.00
Behaviour difficulties	− 0.45 (0.10)	− 0.32***			1.53
Prosocial behaviour	0.38 (0.26)	0.09			1.20
Emotionality	− 0.21 (0.13)	− 0.11			1.28
Step 5					
			0.19	0.01	
Child’s gender	− 2.30 (0.85)	− 0.17			1.07
Child’s age	0.31 (0.30)	0.06			1.00
Behaviour difficulties	− 0.42 (0.11)	− 0.30***			1.58
Prosocial behaviour	0.28 (0.27)	0.07			1.25
Emotionality	− 0.19 (0.14)	− 0.09			1.29
Autistic traits	− 0.06 (0.05)	− 0.11			1.20

*p < 0.05; **p < 0.01; ***p < 0.001

## Discussion

The purpose of the study was to examine the relationships between parental self-efficacy and parental satisfaction on the one hand, and such child characteristics as severity of autistic traits, behavioural difficulties, prosocial behaviour and temperamental characteristics in the parents of autistic children and parents of neurotypical children. The aim of this study was also to evaluate the significance of these characteristics of the child to predict parental self-efficacy and parental satisfaction in parents of autistic children and parents of neurotypical children aged 7–11.

The child characteristics that demonstrated the strongest relationship both with PSE and parental satisfaction in the two groups were behavioural difficulties and prosocial behaviour. What is more, behavioural difficulties and prosocial behaviour in the present study turned out to be significant predictors of PSE and parental satisfaction in parents of autistic children. Behavioural difficulties in children were also the only significant predictor of PSE in parents of neurotypical children.

The relationship between PSE and behavioural difficulties in autistic children (Chen et al., 2021; Rudelli et al., 2021) or in neurotypical children (Albanese et al., 2019; Matalon & Turliuc, 2022) has already been extensively documented. It is hardly surprising, given the fact that difficult behaviour in children is associated with reduced sense of control in parents in everyday situations, tension in social contexts, and anxiety about performing the role of parent (Hodgetts et al., 2013; Myers et al., 2009).

The model tested in this study explained 21% of variation in PSE in parents of autistic children and 3% in parents of neurotypical children as also 27% of variation in parental satisfaction in parents of autistic children and 17% in parents of neurotypical children. This appears to corroborate the opinion of Lievore et al., (2023) that child characteristics have a greater effect on the evaluation of one’s parenting in the parents of autistic children than the parents of neurotypical children. Some authors (Boonen et al., 2014; Giovagnoli et al., 2015) have demonstrated that behavioural difficulties in autistic children may be more impactful for PSE than in neurotypical children, which is likely associated with significantly higher incidence and severity of these problems in the former. Moreover, understanding these behaviours and their causes is a prerequisite for dealing with the child’s behavioural difficulties successfully (Coleman & Karraker, 1998). Mothers of autistic children, compared to mothers of neurotypical children, more often declared that understanding their child’s behavior was difficult for them (Tunali et al., 2002). At the same time, they were aware of the importance of understanding a child’s behaviour and rated its significance higher than mothers of neurotypical children. Even if the parents of autistic children feel effective in their roles, they report the most doubts with respect to their own ability to cope with the behavioural difficulties demonstrated by their children (Huang & Zhou, 2016). What is more, they experience shame due to their child’s behaviours, especially in public, and they feel others are critical of their parenting skills (Gray, 2002). This produces in them not only a sense of guilt, but also of public denouncement and social stigma (Liao et al., 2019), which has significant implications for emotions experienced in relation to parenting. It should be added that the autistic children sample in our study was relatively homogeneous: all children were within the intellectual norm, of similar age and had no comorbid diagnoses. We can therefore assume that, in terms of key characteristics, the group was similar to the group of neurotypical children.

One of the parental tasks is the socialization of the child, i.e., teaching prosocial behaviour and the rules underlying these behaviours and encouraging the child to implement prosocial behaviour (Sanders & Turner, 2018). The child’s openness to the socialization process is one of the predictors of PSE, and parents whose children more often show positive emotions, initiate interactions with parents, and are willing to cooperate or follow parental directions feel more effective in their parental role (Bogenschneider et al., 1997). A review of research by Teague et al., (2017) indicated a negative relationship between the severity of autistic symptoms in children and showing positive emotions to parents, initiating contact with parents, and responding to parents’ behaviour. Additionally, autistic children, compared to neurotypical children, are less likely to respond adequately to another person’s mental state (Baron-Cohen & Wheelwright, 2004) and are less likely to engage in prosocial behaviour, especially in situations that would require reading another person’s emotions or needs by themself (Dunfield et al., 2019; Russell et al., 2012). However, even if the frequency or intensity of prosocial behaviour in autistic children is similar to neurotypical children, parents of autistic children report significantly lower expectations regarding these behaviours (Ryan-Enright et al., 2022). The lower frequency and intensity of prosocial behavior in autistic children and lower expectations of their parents regarding these behaviours make prosocial behaviour significant for parenting and predicting both PSE and parental satisfaction in parents of autistic children.

The results obtained in this study indicate a weak relationship between PSE and such child temperamental traits as emotionality, sociability and shyness, and on top of that—only in the Autistic Group. No such relationships were found in the Neurotypical Group. At the same time, emotionality in children was correlated with parental satisfaction in both groups. The relationships between children’s temperamental traits and parental PSE have been found in previous studies in infants (Lipscomb et al., 2011; Solmeyer & Feinberg, 2011) and toddlers (Giallo et al., 2013; Machida et al., 2002). Obviously, the important factor here may have been the child’s age. Schoolchildren have already developed multiple strategies helpful in the regulation of emotions, mood, and level of stimulation (Eisenberg & Morris, 2002). Moreover, their parents certainly know their children better and understand their behaviour better, plus they are more effective in dealing with the challenges associated with some of their offspring’s temperamental characteristics. It seems plausible, therefore, that temperamental characteristics play a less important role for PSE and parental satisfaction in schoolchildren than it is the case with infants and toddlers.

To the best of our knowledge, there has been no research about the relationships between PSE or parental satisfaction and the severity of autistic traits in neurotypical children. We found a weak negative correlation between these characteristics in neurotypical children and parental satisfaction, but not with PSE. However, it should be noted that in the Neurotypical Group, we included only those children who scored below the cut-off point for the Polish version of AQ-Child. The AQ-Child is a screening tool, and scores above the cut-off point do not indicate that the child could be definitely diagnosed as autistic. This means that only children with relatively low and average severity of autistic traits were included in the Neurotypical Group. Lower autism-related behaviours in children may not be so significant for parents regarding their sense of effectiveness and competence in performing parental tasks. However, they are still connected with the quality of parenting-related emotions.

Previous findings about the relationship between autism symptoms’ severity in autistic children and PSE were inconclusive (Garcia-Lopez et al., 2016; Lindsey & Barry, 2018; Rezendes & Scarpa, 2011; Taylor et al., 2021). Our findings indicate a weak negative correlation between PSE and severity of autistic traits in the Autistic Group. These findings are consistent with the results obtained by Rezendes and Scarpa (2011) or Weiss et al. (2015). In those two studies, the severity of autistic traits was measured with the Social Communication Questionnaire (SCQ; Rutter et al., 2003). SCQ, like The Autism Spectrum Quotient: Children’s Version (AQ-Child) used in our study, is a parent-report questionnaire. In addition, Taylor et al., (2021) demonstrated that the severity of autistic traits in autistic children measured in SCQ was a predictor of PSE. Interestingly, in the same study, when the severity of autistic symptoms in children was evaluated not by parents but by professionals, using the Autism Diagnostic Observation Schedule–Second Edition (ADOS–2; Lord et al., 2012), no significant relationships were found between autistic symptom severity in children and PSE. There have been other reports suggesting a lack of relationship between PSE and autism symptoms severity in children measured with ADOS-2 (e.g. Russell & Ingersoll, 2021). Relationships between the severity of these autism symptoms in children and PSE were also not detected when Childhood Autism Rating Scale (CARS; Schopler et al., 1988) was used. CARS is another tool that focuses on the strengths, severity, and characteristics of the precise symptom features of autism, completed by professionals (Garcia-Lopez et al., 2016).

The general pattern is similar with respect to parental satisfaction. When autism symptoms severity in autistic children was measures using CARS (professional-report instrument), no relationship was found between that variable and parental satisfaction (Tobing & Glenwick, 2007). However, when symptoms of autism were estimated with SCQ (a parent-report measure), the relationship was indeed present (Falk et al., 2014). In our study, the severity of autistic traits in children was also measured using a parent-report questionnaire (AQ-Child) and the results also indicate a negative correlation between that variable and parental satisfaction, both in the parents of autistic children and the parents of neurotypical children. It therefore appears that a significant source of inconsistencies with respect to correlations between the severity of autism symptoms in children and PSE or parental satisfaction lies with who makes the assessment: parent or professional. A similar pattern emerges in the case of parenting stress. The level of that stress is associated with autism symptoms severity as evaluated by parents, but not by professionals (Brei et al., 2015). Thus, parental subjective perception of autism-related behaviours may play a greater role in shaping parenting experience than the actual severity of these behaviours assessed with more objective measures. Lack of longitudinal studies precludes a more comprehensive interpretation of causal relationships between parent’s perception of the severity of child’s autistic traits and PSE or parental satisfaction. However, the lack of support for the relationship between these variables in studies that used more objectivised measures of autism spectrum disorder symptom presence in the child may suggest a direction of intervention that could potentially be offered to parents, as it would seem that a significant factor in the assessment of one’s parenting abilities is the meaning the parent assigns to the child’s behaviour and the way he or she evaluates that behaviour.

### Limitations and Future Directions

The study had several limitations. First, information about the child’s diagnosis was provided by parents only and was not further verified. However, partial confirmation of the diagnosis, or more precisely accuracy of distribution to the group, were results of the AQ-Child. Another limitation of the study was significantly bigger number of mothers than fathers. Future research should focus more on fathers’ perspectives and examine whether there are some differences between mothers and fathers in the relationship between child characteristics and parental self-efficacy or satisfaction. Also, family income level can be significant in PSE or parental satisfaction. Thus, the fact that parental income status was not measured in the study can be considered a limitation. It may also be beneficial to obtain information about the child’s temperament and behavioural difficulties from a source other than a parent-report questionnaire (such us a teacher-report measure or behavioural observation). In addition, the severity of behavioural difficulties, prosocial behaviour and temperamental traits in autistic children were measured using instruments designed for assessing these characteristics in neurotypical children. It remains unclear whether scores obtained using such instruments in neurotypical and their autistic counterparts can be considered equivalent (Barger et al., 2019; Hepburn & Stone, 2006). In the future, it would be useful to evaluate the psychometric properties of such measures with respect to autistic children.

## Conclusions

Severity of the child’s behaviour problems proved to be a significant predictor of PSE and parental satisfaction in the parents of autistic as well as neurotypical children. This may indicate particular usefulness of mental health prevention programs for parents that combine two elements, i.e. developing parents’ abilities of effectively coping with children’s behavioural problems and work on attribution processes and negative convictions about parenthood and the related expectations that make it difficult to evaluate one’s parenthood as satisfying. Programmes of that kind, especially in the cognitive-behavioural paradigm, have already been tested (e.g. Mouten et al., 2018), however, their availability for parents is still unsatisfactory. They still need to be developed in a way that matches the needs of the parents of children of various ages and with different characteristics (difficulty profiles, level of intellectual functioning, communication skills).
